# Current status and solutions for the overuse of emergency CT in pediatric patients with abdominal pain

**DOI:** 10.3389/fped.2025.1654551

**Published:** 2025-09-10

**Authors:** Wei Weng, Yaomeng Chen

**Affiliations:** ^1^ Department of Radiology, The Third Affiliated Hospital of Wenzhou Medical University, Wenzhou, China; ^2^ Department of Radiology, Wenzhou People’s Hospital, Wenzhou, China

**Keywords:** pediatric abdominal pain, emergency CT overuse, radiation exposure risks, defensive medicine, emergency departments, guideline adherence

## Abstract

Pediatric abdominal pain is one of the most common issues reported in emergency departments (EDs), where emergency computed tomography (CT) plays a crucial role in diagnosing various conditions. However, the frequent use of emergency CT scans in children has raised significant concerns due to the associated risks of unnecessary radiation exposure and increased healthcare costs. This review aims to explore the current situation regarding the overuse of emergency CT scans among children with abdominal pain, examining the factors that contribute to this trend and its harmful effects. It also summarizes recommended interventions and research advancements designed to tackle this issue. By thoroughly reviewing the existing literature, this article seeks to provide valuable insights for clinical practice, promoting the careful use of emergency CT and emphasizing the role of non-ionizing alternatives such as ultrasound and magnetic resonance imaging (MRI) to minimize unnecessary radiation exposure and optimize the use of medical resources.

## 1 Introduction

Acute abdominal pain is a frequent issue in pediatric emergency departments (EDs) and often presents a diagnostic challenge due to its complex nature (1). The causes of abdominal pain in children can range from benign conditions, such as functional abdominal pain, to more serious issues like appendicitis or intestinal obstruction (2). The difficulty in diagnosing abdominal pain in young patients is heightened by the variety of symptoms and the overlap between different conditions. This situation underscores the need for effective diagnostic tools that can ensure prompt and accurate treatment. One such tool is emergency computed tomography (CT), which has become a widely used imaging method because of its speed and precision in diagnosing abdominal issues (3).

In recent years, there has been growing concern about the excessive use of emergency CT scans in children who present with abdominal pain (4). Relying too heavily on CT imaging poses significant challenges, especially regarding the exposure of young patients to ionizing radiation, which could increase their risk of developing cancer later in life (5). Furthermore, the financial implications of unnecessary imaging can put additional pressure on healthcare resources, potentially detracting from other vital areas of patient care. Therefore, it is essential to understand the current situation regarding emergency CT use in pediatric cases of abdominal pain, identify the factors that lead to its overuse, and explore possible solutions to improve clinical practices while ensuring the safety and well-being of patients.

The literature indicates that although CT scans can provide important diagnostic information, they are not necessary for every case of abdominal pain in children. Many instances of abdominal pain can be effectively managed through clinical evaluation and other, less invasive imaging methods such as ultrasound or magnetic resonance imaging (MRI), both of which avoid exposing patients to ionizing radiation (6, 7). Therefore, it is crucial to develop clear guidelines and criteria for the appropriate use of emergency CT scans in pediatric settings. This should include assessing clinical factors, laboratory findings, and the availability of alternative imaging options that can deliver similar diagnostic insights without the risks linked to radiation exposure.

Current investigations highlight the importance of a careful approach that weighs the benefits of CT imaging against the risks of radiation exposure, especially for vulnerable populations like children (8). There is a growing emphasis on developing decision-making frameworks that integrate clinical guidelines and scoring systems to help healthcare providers decide when CT scans are truly necessary (9). This strategy could significantly reduce unnecessary imaging, protecting children from excessive radiation exposure while also making better use of healthcare resources.

In recent years, various strategies have been suggested to address the excessive use of emergency CT scans in pediatric patients. These strategies focus on improving the education and training of healthcare professionals about the risks associated with CT imaging, promoting the use of alternative imaging methods, and developing clinical pathways that prioritize conservative management for certain cases of abdominal pain. Additionally, the integration of advanced imaging technologies, like spectral CT, could provide enhanced diagnostic capabilities while minimizing radiation exposure, indicating a hopeful avenue for future research and clinical application (10).

In summary, as pediatric emergency medicine evolves, it is crucial to critically evaluate the role of emergency CT scans in managing pediatric patients. Addressing abdominal discomfort in children poses considerable challenges. By addressing concerns about overuse and exploring innovative strategies, we can improve patient safety, enhance clinical outcomes, and ensure the responsible use of medical resources in pediatric healthcare settings.

## 2 Overuse of emergency CT in pediatric abdominal pain: evidence, causes, and solutions

### 2.1 Current application of emergency CT in the diagnosis of abdominal pain in children

In recent years, the deployment of emergency CT scans in pediatric patients presenting with abdominal pain has notably escalated, particularly among those exhibiting non-specific abdominal discomfort (11). This trend prompts apprehensions regarding the suitability of CT application, especially since numerous instances lack definitive clinical justifications for such imaging. Evidence from diverse healthcare facilities reveals that the incidence of emergency CT scans can attain concerning levels (12). The excessive dependence on CT imaging is particularly alarming when one considers the associated risks posed by ionizing radiation, particularly in pediatric patients who are inherently more vulnerable to the detrimental effects of radiation exposure. A significant fraction of these scans resulted in normal findings, suggesting that numerous children were unnecessarily exposed to radiation without any substantial diagnostic advantage.

Moreover, the rising trend in CT usage can be attributed to several factors, such as the increasing reliance on advanced imaging technologies in clinical decision-making, the urgency to accelerate diagnoses in emergency contexts, and the potential inclination towards defensive medicine practices among healthcare practitioners. The ramifications of these patterns are significant, as they not only lead to heightened healthcare expenditures but also evoke ethical dilemmas concerning patient safety and the prudent allocation of medical resources. Consequently, there exists an urgent necessity for the formulation and enactment of clinical guidelines that advocate for a more selective application of CT scans in pediatric patients presenting with abdominal pain, ensuring that imaging is reserved for scenarios where it is clinically warranted and essential for effective management (13, 14)

### 2.2 Definition and assessment criteria for overuse of emergency CT

#### 2.2.1 Definition of overuse

In medical diagnostics, especially in emergency care settings, “overuse” refers to the unnecessary application of diagnostic procedures like CT scans when there are no clear clinical indications or when other assessments could adequately guide clinical decisions (15). For instance, administering CT scans in situations where the risks, such as exposure to ionizing radiation and increased healthcare costs, outweigh the clinical benefits exemplifies overuse. This concern is particularly significant for pediatric patients, as children are more susceptible to the harmful effects of radiation, potentially leading to long-term consequences like an increased risk of cancer later in life (16). Studies show that the overutilization of diagnostic imaging, including CT scans, is prevalent across various healthcare settings, resulting in higher healthcare costs without improving patient outcomes (17). Overuse can be divided into two main categories: the first involves opting for a CT scan even when there are alternative, less invasive diagnostic options available, while the second pertains to the repeated use of CT scans that do not provide any additional clinical value. The latter is especially troubling, as it can lead to unnecessary radiation exposure and increased healthcare spending without enhancing diagnostic accuracy or patient care (18).

The consequences of overusing medical imaging extend beyond the safety of individual patients and highlight broader systemic issues within the healthcare system. For example, when CT scans are overused, they can trigger a cascade of additional tests and procedures, which not only places extra pressure on healthcare resources but also worsens existing inefficiencies (19). Moreover, the lack of standardized guidelines and clinical decision support tools (CDSTs) often leads to inconsistent practices among healthcare providers, resulting in varied application of imaging protocols (20) Addressing the problem of overuse requires a well-rounded approach that includes educating healthcare professionals about appropriate imaging practices, implementing clinical decision support systems, and fostering a culture of collaborative decision-making with patients (21) Ultimately, it is essential to establish a clear and consistent definition of overuse, along with targeted interventions, to optimize diagnostic practices and ensure patient safety, especially in emergency medical situations.

#### 2.2.2 Assessment criteria and related research

The analysis of CT utilization in pediatric EDs, particularly concerning abdominal pain, is crucial for evaluating the appropriateness of imaging practices. Various studies have highlighted important metrics such as CT utilization rates, positive detection rates, and the consistency of clinical decision-making with established guidelines (22). For instance, a retrospective study examining an appendicitis evaluation algorithm found that CT usage did not significantly decrease after the guidelines were implemented, remaining around 25.7% to 24.8%. Furthermore, the median length of stay in the emergency department (ED) increased from 333.5 to 362 min (23). This suggests that even with the introduction of guidelines, the frequency of CT scans remains high, indicating potential overutilization of this imaging method. Additionally, the positive detection rate for appendicitis did not show significant improvement, raising concerns about the effectiveness of current protocols in minimizing unnecessary imaging and the associated healthcare costs.

The appropriateness criteria set by the American College of Radiology (ACR) serve as a benchmark for evaluating the need for imaging in pediatric patients (24). A study examining the effectiveness of these criteria revealed that a significant number of CT scans performed for suspected appendicitis did not align with the recommendations, particularly in patients with low Appendicitis Inflammatory Response (AIR) scores (25). This finding underscores the importance of strictly following clinical guidelines to mitigate the risks linked to radiation overexposure and to prevent unnecessary surgical interventions. Additionally, the study found that while CT scans were often the preferred imaging choice, ultrasound was still recommended as a diagnostic option, particularly in cases with high AIR scores. These results emphasize the need for incorporating clinical decision-making frameworks that prioritize patient safety and adhere to evidence-based guidelines.

In addition to the ACR guidelines, research has explored how consistent clinical decision-making is among emergency physicians when it comes to ordering CT scans. A survey conducted with emergency medicine practitioners indicated that defensive medicine, driven by the fear of malpractice, significantly impacts their imaging decisions, leading to a rise in unnecessary CT scans (26). This issue is further intensified by the pressures from patients and the fast-paced environment of EDs, where quick decision-making is essential. The findings revealed that both radiologists and emergency physicians recognize the overuse of CT scans as a major concern, with these scans making up a substantial portion of imaging overuse in emergency settings. This highlights the urgent need for educational initiatives that aim to reduce unnecessary imaging practices and foster a culture of evidence-based decision-making within emergency medicine.

A systematic review focused on the overuse of CT scans in children with mild head injuries highlighted that following established guidelines, like the Canadian CT Head Rule (CCHR), could significantly reduce unnecessary imaging (27). The review found that a considerable number of CT scans performed did not meet the necessary criteria, indicating a clear opportunity for improvement in clinical practice. These findings suggest that enhancing training and raising awareness of existing guidelines among ED staff could lead to better patient outcomes and lower healthcare costs.

This evidence highlights persistent challenges in aligning imaging practices with established protocols. Strengthening adherence to guidelines and implementing decision-making frameworks will be key to improving patient safety and reducing unnecessary imaging.

#### 2.2.3 Existing research on overuse data

Numerous studies have highlighted a concerning trend regarding the overuse of CT scans in children presenting with abdominal pain, indicating that many scans may not be clinically necessary (14). Surveys of radiologists and emergency physicians have identified CT as the imaging modality most commonly associated with overuse, with a significant proportion of respondents attributing this to defensive medical practices (26).

Evidence from other clinical contexts also supports this concern. For instance, a systematic review of CT use for mild head injuries reported an overutilization rate of approximately 27%, with wide variation depending on the criteria applied (28). Similarly, although validated tools such as the Pediatric Emergency Care Applied Network (PECARN) rule and the CCHR exist to guide clinical decision-making, their application remains inconsistent across institutions, leading to unnecessary imaging in children (27, 29).

Empirical studies further demonstrate the scale of this problem. One investigation found that 93.6% of CT scans performed for pediatric minor head trauma yielded negative findings, underscoring the risks of unnecessary radiation exposure and the associated financial burden (30). Likewise, a quality improvement project targeting appendicitis diagnosis reduced CT utilization from 31.3% to 12.1% without adverse effects on patient outcomes (31), showing that structured interventions can effectively curb overuse.

The literature provides clear evidence of substantial CT overuse in pediatric patients. Consistent adherence to validated decision-making tools such as PECARN and CCHR is needed to minimize unnecessary radiation exposure and improve resource utilization (28, 30).

### 2.3 Potential reasons for overuse of emergency CT

#### 2.3.1 Clinical decision-making factors for clinicians

Clinical decision-making in pediatric emergency settings is often influenced by a combination of psychological, clinical, and systemic pressures. Physicians may lack confidence in clinical evaluations, particularly when symptoms are ambiguous and the stakes of a missed diagnosis are high. For instance, in suspected appendicitis, the urgent need for a rapid and accurate diagnosis can lead clinicians to favor CT imaging, despite concerns about radiation exposure in children (32).

Time constraints in busy EDs further reinforce this tendency, as CT offers immediate results compared with more detailed clinical assessments. In such high-pressure situations, the urgency to act can overshadow considerations of appropriateness, leading to unnecessary imaging.

Importantly, this pattern is compounded by the fear of malpractice liability. As noted in Section 2.2.3, survey data identified defensive medicine as a major driver of CT overuse. Here, we expand on the clinical reasoning behind this phenomenon: physicians may order CT scans preemptively to protect themselves against potential legal repercussions from missed diagnoses, even when clinical guidelines recommend more conservative approaches (33).

Together, these factors explain the frequent overuse of CT in pediatric abdominal pain. More robust guidelines and decision-support tools are needed to reduce reliance on CT while safeguarding patient safety.

#### 2.3.2 Factors related to patients and families

Managing abdominal pain in children often requires significant input from parents regarding the urgency of diagnosis. Many parents, driven by anxiety over their child's health, may request CT scans even when not clinically indicated. Studies have shown that parents often underestimate the risks of radiation exposure, particularly in repeated imaging, which is a common concern in pediatric care (34). This parental anxiety can cloud judgment and increase demand for unnecessary CT imaging, sometimes conflicting with best clinical practices (35).

#### 2.3.3 The influence of healthcare systems and policies

The framework of healthcare systems and policies plays a crucial role in shaping the use of medical imaging, particularly with respect to CT scans for pediatric patients experiencing abdominal pain. A significant contributor to the excessive application of CT scans is the apprehension surrounding medical malpractice litigation, which fosters defensive medical practices. Healthcare professionals frequently opt for advanced imaging modalities such as CT scans as a precautionary tactic to avert potential legal consequences, even when clinical guidelines might recommend alternative approaches. This defensive strategy not only escalates healthcare expenditures but also subjects young patients to unnecessary radiation exposure, a particularly alarming concern given their heightened sensitivity to the detrimental effects of radiation compared to adults (36). In addition, the lack of standardized clinical guidelines or a consensus regarding the appropriate utilization of CT scans intensifies the dilemma. The variability in practice patterns across various healthcare facilities and geographical regions results in inconsistent application of imaging protocols, leading to some patients receiving excessive imaging while others may not undergo essential evaluations. The absence of cohesive guidelines can generate confusion among healthcare providers about the suitable indications for CT scans, thus contributing to their overuse (37). Furthermore, economic incentives within healthcare organizations can exacerbate the issue. Financial motivations may drive an increased reliance on imaging services as a means of generating revenue, thereby perpetuating the cycle of overutilization. Institutions may prioritize financial gains from imaging services over patient-centered care, resulting in a disconnect between clinical necessities and imaging practices. This economic pressure can compel healthcare providers to request additional imaging studies, even when such actions may not be clinically justified (38). Tackling these challenges necessitates a comprehensive strategy that includes the establishment of clear, evidence-based clinical guidelines, educational programs for healthcare professionals, and policy changes that align financial incentives with patient safety and high-quality care. By cultivating a healthcare milieu that emphasizes appropriate imaging practices, we can reduce the risks associated with the overutilization of CT scans in pediatric patients and improve the overall quality of care provided.

In addition, it is important to recognize that the extent of CT overuse varies among regions depending on existing regulatory frameworks (39). For instance, health systems that implement auditing mechanisms, prior authorization requirements, or regular monitoring of imaging practices have reported lower rates of unnecessary CT utilization (17). These policy-driven approaches serve as external checks that encourage adherence to guidelines and discourage defensive or profit-driven overuse of diagnostic tools. Conversely, in settings where such oversight is limited, variability in physician decision-making is more likely to result in overutilization (26). Incorporating these regional and policy considerations provides a more comprehensive understanding of the factors influencing CT use in pediatric emergency care.

### 2.4 Adverse effects of emergency CT overuse

#### 2.4.1 Risks of radiation exposure to children

Children are particularly sensitive to radiation exposure due to their developing tissues and longer life expectancy, which increases the likelihood of health risks related to radiation appearing later in life. This heightened sensitivity is largely attributed to the rapid cell growth that occurs in children, making them more vulnerable to the damaging effects of ionizing radiation. This concern is especially significant in the realm of diagnostic imaging, where procedures such as CT scans are commonly used. Research has shown a link between cumulative radiation exposure and a higher risk of certain cancers, notably leukemia and thyroid cancer, in children (40). For instance, studies have found that even low doses of ionizing radiation can significantly raise the risk of leukemia in children, with evidence suggesting that the risk increases with the number of CT scans a child undergoes (34, 41). This relationship highlights the importance of exercising caution when it comes to using diagnostic imaging for young patients. Furthermore, the connection between radiation exposure and thyroid cancer has been well-documented, particularly among children who have been exposed to radioactive iodine following nuclear accidents (42).

Monitoring cumulative radiation exposure from medical imaging is crucial, as research shows that even low levels of radiation can lead to significant long-term health effects, especially in children who may have multiple imaging tests during their early years (43). For instance, a study examining children who underwent imaging for various medical conditions found that those who had four or more CT scans before the age of six faced a significantly higher risk of developing cancer compared to those who had fewer scans (44). This highlights the importance of exercising caution when using radiation-based imaging techniques, particularly for pediatric patients, to minimize potential health risks.

Efforts to minimize radiation exposure involve implementing strict guidelines for imaging procedures, notably the As Low As Reasonably Achievable (ALARA) principle, which emphasizes reducing radiation doses while ensuring diagnostic accuracy (45). Additionally, advancements in imaging technology, including the development of low-dose CT protocols and the use of alternative imaging techniques such as ultrasound and MRI, can significantly reduce reliance on ionizing radiation in pediatric diagnostics (46, 47).

Healthcare professionals should engage in thorough risk-benefit discussions with patients and their families about the necessity of imaging procedures that involve radiation. By informing parents and caregivers about the potential dangers associated with radiation exposure, they can make informed decisions regarding their children's medical care (45). Although imaging is crucial in pediatric diagnostics, awareness of radiation risks and strategies that prioritize safety must remain central to clinical practice.

#### 2.4.2 Medical resource waste

The unnecessary conduct of medical examinations, particularly the overreliance on CT scans for pediatric patients experiencing abdominal discomfort, presents a significant challenge in emergency medicine. This practice not only drives up healthcare costs but also leads to the inefficient use of emergency resources, ultimately putting at risk the care of patients who truly need immediate attention. The financial impact of such overutilization is substantial; studies show that unnecessary imaging contributes to billions of dollars in wasted healthcare spending annually, with estimates indicating that around 30% of total medical expenses could be deemed wasteful or unnecessary (48). Furthermore, the heavy reliance on advanced imaging techniques like CT scans can trigger a cascade of additional diagnostic procedures, which not only burden healthcare systems but also expose patients to unnecessary radiation and potential psychological stress from false-positive results. This issue is especially critical in pediatric populations, where the risks associated with radiation exposure are heightened due to their developing tissues and longer life expectancy, which may increase their susceptibility to future cancers (49).

In EDs, the pressing need to quickly diagnose and treat patients can create a tendency to rely heavily on imaging as the main diagnostic method, often without adequate clinical justification. This tendency is often driven by the fear of missing serious conditions, such as appendicitis or other acute abdominal problems, which can result in severe complications if not identified swiftly. However, an overreliance on CT scans can detract from the essential role of thorough clinical evaluations and history-taking, which are crucial for guiding appropriate diagnostic decisions. Additionally, incorporating CDSTs and following established guidelines can help reduce unnecessary imaging by providing evidence-based recommendations on when imaging is truly needed (50).

The consequences of overutilization in healthcare go beyond just financial strain; they also lead to significant inefficiencies within healthcare systems. For example, when patients face long waiting times for imaging services, it can create bottlenecks that slow down patient flow and delay necessary treatments for those who truly need care. This situation can create a harmful cycle where the demand for imaging increases, putting additional pressure on resources and resulting in more unnecessary tests being ordered to cope with the backlog (51). To address these issues, it is crucial for healthcare professionals to participate in ongoing education about the responsible use of imaging. This education should emphasize the importance of delivering high-value care that focuses on patient safety and effective resource management.

The implementation of strategies like value-based care models can shift the focus from merely increasing the volume of services to optimizing patient outcomes. This transformation can be supported by initiatives that promote shared decision-making between clinicians and patients, ensuring that imaging is used wisely and only when it adds significant value to the patient's diagnostic and treatment plan (52). Reducing unnecessary CT scans is therefore important not only for lowering costs but also for improving the quality and sustainability of pediatric emergency care.

### 2.5 Solutions to reduce overuse of emergency CT scans

#### 2.5.1 Application of CDSTs

The integration of CDSTs in pediatric emergency medicine has become essential for enhancing the careful use of diagnostic imaging, especially CT scans (20). A significant development in this field is the use of artificial intelligence (AI) to develop clinical decision systems that assist healthcare providers in assessing the need for CT imaging in children who present with abdominal pain. These AI-driven systems can evaluate patient data, clinical guidelines, and past outcomes to provide evidence-based recommendations, which helps reduce unnecessary CT scans and the associated risks of radiation exposure. For instance, a recent study demonstrated that using an AI-enhanced CDST significantly lowered the rate of unnecessary imaging in pediatric patients, leading to better patient safety and reduced healthcare costs (53). Furthermore, these tools can simplify the decision-making process, enabling clinicians to focus more on immediate patient care rather than navigating complex diagnostic pathways.

The introduction of clinical decision rules, such as the Pediatric Appendicitis Score (PAS), alongside AI systems, has effectively reduced unnecessary CT examinations. These scoring systems provide a structured way to evaluate the likelihood of appendicitis based on various clinical factors, assisting clinicians in their diagnostic processes. By using these clinical decision rules, healthcare providers can better categorize patients according to their risk levels, leading to a more thoughtful use of imaging techniques. Research indicates that utilizing the PAS not only decreases reliance on CT scans but also enhances the accuracy of appendicitis diagnoses, ultimately improving patient outcomes (54). The incorporation of these decision-making frameworks into clinical practice represents a significant step forward in managing pediatric abdominal pain, ensuring that imaging is conducted only when truly needed.

The successful implementation of CDSTs requires overcoming certain challenges, including alert fatigue and acceptance among providers. Clinicians may become numb to the alerts generated by these systems, which can lead to a diminished response to important recommendations. Therefore, it is crucial to design these tools with a focus on the users, ensuring that alerts are relevant to the context and actionable. Engaging healthcare providers in the development and ongoing refinement of these systems can improve their usability and foster a culture of collaborative decision-making (55). Moreover, continuous education and training on the effective use of CDSTs can empower clinicians to use these tools with confidence, ultimately enhancing adherence to clinical guidelines and improving patient care.

CDSTs, especially those integrating AI and decision rules, show strong potential for reducing unnecessary CT use and improving patient safety. Future research should refine these systems and evaluate their long-term impact on pediatric outcomes.

#### 2.5.2 Strengthening physician training and guideline promotion

As highlighted in Section 2.2.2, current evidence indicates that adherence to established guidelines such as the ACR criteria remains insufficient, contributing to unnecessary CT utilization. Therefore, strengthening physician training and promoting guideline implementation are essential solutions. Targeted educational initiatives, continuous professional development, and dissemination of guidelines through workshops, online platforms, and clinical reminders can improve awareness and compliance. Involving key stakeholders such as pediatricians and radiologists in adapting guidelines to clinical practice will further enhance their acceptance. This combined approach can reduce practice variability, optimize imaging decisions, and ultimately improve both patient safety and healthcare resource utilization (56, 57).

#### 2.5.3 Doctor-patient communication and parental education

As discussed in Section 2.3.2, parental anxiety and limited awareness of radiation risks are important factors contributing to the overuse of CT imaging. To address this issue, effective communication between healthcare professionals and parents is essential. Physicians should provide transparent information about radiation risks and emphasize safer alternatives, such as ultrasound or MRI. This approach not only reduces parental anxiety but also empowers families to participate meaningfully in decision-making regarding their child's care (58, 59).

In addition to factual information, it is crucial to acknowledge the emotional dimensions of parental concerns. Many parents experience guilt or anxiety about the potential long-term effects of radiation exposure on their children. By practicing empathetic communication strategies—such as active listening and validating parents' worries—clinicians can create a supportive environment that facilitates open dialogue. This is particularly valuable in emergency settings, where rapid decisions are often required. Training healthcare professionals in communication skills, including the use of plain language to explain complex medical concepts, can further improve these interactions (60, 61).

Educational tools such as visual aids, brochures, or digital resources can enhance parents' understanding and retention of medical information. When families are thoroughly informed about both the risks and the benefits of CT imaging, as well as the availability of non-ionizing alternatives, they are more likely to feel confident in their decisions. This process should also involve discussion of the cumulative effects of repeated imaging, underscoring the importance of using CT only when truly necessary.

Strengthening doctor–parent communication and providing targeted education are key to reducing unnecessary CT use. Shared decision-making can ease parental concerns, improve trust, and enhance both patient safety and family satisfaction (62, 63).

#### 2.5.4 Optimization of alternative examination methods

Ultrasound has become an important alternative to CT for evaluating abdominal pain in children, primarily due to its non-invasive nature and the absence of ionizing radiation (64). This is particularly beneficial for children, who are more vulnerable to the harmful effects of radiation. When appendicitis is suspected, studies have shown that ultrasound can effectively visualize the appendix and identify signs of inflammation, such as fluid buildup or peri-appendiceal abscesses, with sensitivity and specificity comparable to CT imaging (65). Additionally, ultrasound is readily available in most EDs and can be performed at the bedside, allowing for quick assessment and diagnosis. However, it is important to recognize that while ultrasound is a valuable diagnostic tool, its effectiveness can depend on the operator's expertise, and in some complex cases, it may not provide sufficient detail to rule out serious conditions. Therefore, clinicians should remain vigilant and consider further imaging, such as CT, if ultrasound results are unclear or if the clinical situation suggests a more serious underlying issue (66).

MRI offers a valuable non-radiative alternative to CT in certain clinical scenarios, especially for pediatric patients experiencing abdominal pain. It excels in providing excellent soft tissue contrast and detailed images of abdominal organs without exposing patients to ionizing radiation (7). This feature is particularly beneficial when there are concerns about conditions like mesenteric ischemia or complex abdominal masses, where accurate anatomical details are crucial for effective management (67). However, the use of MRI in acute situations can be limited by factors such as availability, longer imaging times, and the need for sedation in younger children. Fortunately, recent advancements in MRI technology, including faster imaging sequences and improved sedation protocols, have made it more feasible for use in emergency settings. Moreover, MRI is especially advantageous for cases requiring repeated imaging, as it eliminates the cumulative radiation risk associated with multiple CT scans (68).

CT remains essential for rapid diagnosis, but ultrasound and MRI offer important advantages without radiation exposure. Integrating these alternatives into practice will be key to improving safety and diagnostic accuracy in pediatric care (69). When CT is unavoidable, advances in low-dose technology, as discussed in Section 2.6.1, may help mitigate radiation risks.

### 2.6 Future research directions and challenges

#### 2.6.1 Application of low-dose CT technology

The emergence of low-dose CT technology represents a significant advancement in medical imaging, particularly for pediatric populations where concerns about radiation exposure are paramount. Recent studies have shown that innovative low-dose CT techniques can effectively reduce radiation exposure while maintaining diagnostic accuracy. For instance, the application of tin (Sn) filters in CT imaging has been proven to lower radiation doses by over 90% without compromising image quality, especially in cases like attenuation correction for positron emission tomography (PET) scans (70). Additionally, the development of ultra-low dose CT protocols has been validated through extensive phantom studies, which demonstrated that these protocols can achieve diagnostic image quality comparable to that of standard dose CT, thereby fostering safer imaging practices for children (71). Moreover, the integration of advanced reconstruction algorithms, including deep learning techniques, has further enhanced the quality of low-dose CT images by effectively reducing noise and improving spatial resolution (72). These advancements not only decrease the risk of radiation-induced complications in children but also support a significant shift towards the routine adoption of low-dose imaging protocols in clinical environments, particularly for conditions that necessitate frequent imaging, such as assessments of abdominal pain in pediatric EDs.

The comparative assessment of low-dose CT against traditional imaging techniques has shown promising results. For example, a study focused on evaluating renal colic in children found that low-dose CT significantly reduced radiation exposure while still providing reliable diagnostic information. This aligns with the goal of minimizing unnecessary radiation in vulnerable populations (73). The ability to obtain high-quality images with lower radiation doses is especially relevant given the growing public health concerns about the long-term effects of radiation exposure, particularly in children. As healthcare professionals continue to seek ways to optimize imaging protocols, the use of low-dose CT technology stands out as a practical solution that balances the need for accurate diagnostics with the essential aim of protecting patients from the potential risks of ionizing radiation.

Low-dose CT technology enhances patient safety and encourages a more responsible approach to imaging. Continued research in this area will be essential for safer and more effective pediatric diagnostics.

#### 2.6.2 AI-assisted diagnosis

AI has emerged as a groundbreaking instrument in the field of medical diagnostics, particularly concerning the assessment of pediatric patients who present with abdominal pain (74). Beyond current CDST applications mentioned in Section 2.5.1, future research should focus on expanding AI's role in risk stratification and personalized diagnostic pathways. The incorporation of AI algorithms within clinical workflows presents a promising pathway for improving diagnostic precision and reducing the subjective biases that are inherently present in human decision-making (75). For example, research has shown that AI models can attain high sensitivity and specificity levels in identifying conditions such as appendicitis or intussusception, which require prompt attention in pediatric populations (76). Additionally, AI can play a role in categorizing patients based on their risk profiles, enabling customized diagnostic strategies that adhere to clinical best practices and guidelines. For instance, AI-enabled tools can identify patients exhibiting specific clinical signs that necessitate immediate imaging, while also proposing alternative management options for those presenting with lower-risk conditions (77). The adoption of AI-assisted diagnostic frameworks not only enhances the efficiency of clinical workflows but also possesses the potential to improve patient outcomes by promoting timely and precise diagnoses. Nevertheless, it is crucial to acknowledge the limitations of AI, which include the necessity for comprehensive training datasets and the critical role of clinician oversight to ensure that AI recommendations are consistent with clinical judgment (78). As AI technology continues to advance, ongoing research and validation will be vital to fully harness its potential in pediatric emergency medicine and to solidify its status as an essential element of diagnostic decision-making.

#### 2.6.3 Exploration of multidisciplinary collaboration models

The collaboration across various disciplines in the healthcare sector, especially within emergency contexts, has become an essential approach for improving patient outcomes, particularly in intricate situations such as abdominal pain in pediatric patients. The amalgamation of specialized knowledge from pediatrics, radiology, and emergency medicine can significantly enhance the diagnostic workflow, curtail unnecessary imaging procedures, and elevate the overall care trajectory for children experiencing abdominal discomfort. For example, a well-coordinated multidisciplinary team can promote prompt decision-making by ensuring that all pertinent clinical viewpoints are taken into account, thereby optimizing resource allocation and minimizing redundancies in treatment. Research indicates that productive teamwork among healthcare professionals fosters a clearer comprehension of patient requirements, not only improving the quality of care but also shortening hospital stays and decreasing the likelihood of complications (79). In the realm of pediatric emergency care, where abdominal pain frequently arises, the contribution of radiology assumes particular importance. By engaging radiologists early in the diagnostic sequence, teams can judiciously apply imaging techniques, ensuring that CT scans are utilized only when genuinely necessary. This consideration is particularly pertinent in light of the concerns regarding radiation exposure in children and the risks associated with the excessive use of imaging technologies, which could result in increased healthcare expenditures and unwarranted anxiety for families (80). Additionally, multidisciplinary collaboration nurtures a culture of shared accountability, wherein team members are motivated to communicate candidly about their insights and apprehensions relating to patient care. Such a cooperative atmosphere can improve the precision of diagnoses and the suitability of treatment strategies, ultimately yielding better health outcomes for pediatric patients (81). Nonetheless, obstacles persist in the realization of effective multidisciplinary collaboration, including the necessity for well-defined communication pathways, clarified roles within the team, and a commitment to collective objectives. Tackling these challenges is vital for the effective incorporation of multidisciplinary methodologies in EDs, particularly in refining the management of pediatric abdominal pain (82). As healthcare systems increasingly acknowledge the significance of collaborative care frameworks, continuous research and training are imperative to enhance these methods and ensure their practical application. In summary, the investigation of multidisciplinary collaboration frameworks in emergency settings presents substantial potential for improving both the diagnostic and treatment processes for children suffering from abdominal pain, ultimately leading to superior patient care and more efficient resource use.

#### 2.6.4 Development of pediatric-specific imaging guidelines

The imaging protocols used for pediatric patients, especially those with abdominal pain, largely depend on data from adult populations. This reliance creates a significant gap because children have unique physiological characteristics. There is an urgent need to establish standards specifically for pediatrics that consider these differences, particularly since conditions like appendicitis can present differently in younger patients, requiring tailored imaging approaches. Additionally, concerns about radiation exposure from imaging techniques, such as CT, highlight the necessity for guidelines that prioritize both safety and effectiveness for children. These guidelines should be developed through collaboration among healthcare professionals and the involvement of families. Creating imaging guidelines focused on pediatric needs could improve diagnostic accuracy, enhance clinical outcomes, and minimize unnecessary radiation exposure for children experiencing abdominal pain and other acute medical conditions (83–85).

## 3 Conclusion

The overreliance on emergency CT scans in pediatric patients with abdominal pain is a multifaceted issue that requires careful consideration from various clinical, patient, and systemic perspectives. This review emphasizes the complexities involved in this challenge, revealing that the factors contributing to the excessive use of CT scans are not solely clinical in nature. Instead, they are influenced by patient expectations, systemic pressures, and the availability of alternative diagnostic methods.

From a clinical standpoint, the preference for CT imaging frequently arises from a combination of diagnostic uncertainty and the pressing need to improve patient management quickly. Emergency physicians, faced with the challenge of making swift decisions in high-stress environments, may feel compelled to use CT scans to rule out serious medical conditions. As discussed earlier, children's heightened sensitivity to radiation underscores the importance of minimizing unnecessary CT exposure (86).

The challenge of overutilization in healthcare is further complicated by systemic factors, particularly the pressures of healthcare delivery systems that prioritize quick turnover and operational efficiency. In such environments, there is a tendency to rely on imaging techniques that provide immediate results, often sacrificing more careful and considered methods. This reliance not only increases unnecessary radiation exposure for patients but also leads to the misallocation of valuable healthcare resources, shifting attention and funding away from more effective, evidence-based practices.

To address these challenges, it is essential to promote the creation of clinical guidelines tailored for pediatric patients. These guidelines should be developed through a collaborative approach that includes input from pediatricians, emergency medicine specialists, radiologists, and other relevant stakeholders. Furthermore, improving medical training and education regarding the careful use of imaging in pediatric abdominal pain cases can foster a culture of critical thinking and thoughtful decision-making. This involves highlighting the importance of thorough clinical evaluations and considering alternative diagnostic methods, such as ultrasound or MRI, which pose significantly lower radiation risks (87).

The development of decision support systems is crucial in reducing the excessive dependence on CT scans. These systems provide clinicians with evidence-based guidance and real-time information, helping them make informed decisions that balance the need for quick diagnoses with the responsibility to minimize unnecessary radiation exposure. Additionally, incorporating these systems into electronic health records enhances their usability and ensures they are readily available at the point of care (88).

Future research should focus on developing pediatric-specific guidelines that address the unique characteristics of this age group. This involves exploring the effectiveness of different interventions aimed at reducing the overuse of CT scans and assessing how educational programs can impact clinician practices and patient outcomes. Longitudinal studies that track the implementation of these strategies will be vital in confirming their effectiveness and improving clinical practices.

In summary, the excessive use of emergency CT scans in children with abdominal pain is a complex issue that requires a comprehensive solution. By integrating various perspectives from clinical practice, patient needs, and systemic factors, we can move towards a more effective management strategy. It is crucial to apply imaging techniques judiciously, prioritizing patient safety while optimizing healthcare resources. Looking ahead, the emphasis should be on collaboration, education, and innovation to ensure that our diagnostic methods are not only effective but also responsible in safeguarding the health of our youngest patients.
